# Annotations

**Published:** 1890-11-22

**Authors:** 


### ANNOTATIONS.

"Charlotte Martin, Hospital Nurse."

In the *British Weekly* there is now being published a series of "London Autobiographies." The second article of the series appeared last week, and purported to be the autobiography of Charlotte Martin, hospital nurse. It is a sober article, and evidently written by an experienced nurse, who understands and values her profession. Questions of nursing we usually deal with in the "Nursing Mirror," but certain points are raised by Miss Martin which should be considered by hospital managers and the public, as well as by nurses. The recent outcry about the harsh and slavish treatment of nurses at hospitals has excited a good deal of public feeling. Those who are familiar with the inner working of hospitals know how to discount what has been advanced by the enemy; but the public can only judge by what it hears or reads; and it is well known that with inexperienced hearers the noisiest advocate generally secures the largest number of votes. Miss Martin admits the strenuousness of a nurse's work in general, and expresses judgments about matrons as a class which they would do well to take to heart. But she enables us to perceive quite clearly the origin of many of the tales of hardship and slavery. Not the tyranny of the matron or the indifference of the committee is the commonest cause of unhappiness in probationer nurses, but their own incompetency and want of even the rudiments of common sense. "There are incompetent nurses," says Miss Martin "as there are incompetent persons in every profession. But in the London hospitals these are soon weeded out. I called some weeks ago at a West-end hospital to enquire about an old school friend of mine who, I heard to my astonishment, had taken to nursing. She had been living for five years in a German town, going three times a-week to the theatre, and had been at least three times engaged to be married. She had no family ties and no special interests, so as a last dissipation she had tried hospital nursing. I went to the matron's room to ask for her, but found that she had only stayed in the hospital three months, and had shown herself hopelessly incompetent." Miss Martin's experience of "incompetents" is much more favourable than the writer's. Within a month of the present time the "Experiences of a Nurse" were offered for publication, in which a detailed story of harrowing illusage was both the pudding and the seasoning. How long had this particular nurse been subjected to the tyranny of the matron and the neglect of the committee? For two whole days—not two hours, but two entire days. At the end of that time this young woman was thoroughly qualified to lead a new crusade against hospitals and to expose every weak spot in their armour with the most absolute accuracy. We steadily uphold the rights of women in these pages; but we know well to what depths of hopeless inconsequence many women can plunge themselves in a single moment. Those who strive to reach the truth concerning hospitals and their nurses will do well to bear in mind that there are women and women. Since Solomon's days, and before, a good woman has been a crown of glory to her family; whilst a foolish woman has been one of the very unhappiest and most unendurable of all the afflictions which a world of probation has produced. Hospitals, which employ such a large number of women, have often proved this to their cost.

Hospital Chaplains.

An Irishnurse, who was trained in Dublin, but who has for some time been working in London, expresses the opinion that the chaplains at the Metropolitan Hospitals are a very indifferent set of persons. The nurse herself is a Protestant, and is said to be one of the best women in a sick room to be found in London. She is of opinion that great reforms are needed. The chaplains, she says, are usually old and broken-down clergymen, who perform their duties in a dull and perfunctory way, and have no real sympathy with the patients. It grieves her sometimes to see the eagerness with which Roman Catholic patients drink in the words of their priests—how they cling to them and will hardly let them out of their sight; while Protestants turn away coldly from the ministrations of their chaplains, as if they felt them to be prompted by duty rather than by love. It is impossible for us to say to what extent this picture represents the actual facts. If it be the rule, it is a rule to which there are not a few exceptions. But if it be the rule, it is a disgrace to our Reformed religion, and a scandal in the eyes of all right-minded persons. If any class of people need sympathy it is those who are sick with severe diseases; and most hospital patients are in this condition. And if all sick persons need to be comforted, surely those need most who have been taken away from home and friendly faces to be surrounded by strangers, and so to a certain extent to endure their sufferings alone. The Catholic religion has not in itself more of comfort than the Protestant; for in their essence the two are the same. But comfort and help are often more in the minister than in his doctrines. The doctrines, indeed, are mere words without life if the minister be destitute of faith and love. Duty is a great word; and an iceberg is a great thing, but it has a wonderful degree of chilling power. It is much to be feared that there is a great deal of truth in what this warm-hearted Protestant Irishwoman says about the perfunctory character of the religious ministrations in many of our hospitals. It is a cruel thing to deprive the sick of religious consolation; but still more cruel to offer them a semblance of consolation, from which they cannot but turn wearily and disappointedly away. What have hospital committees to say upon the subject? They must rouse themselves to a sense of duty in this as in other serious hospital affairs. Every member of a London committee must hold it his duty to make sure that at his particular hospital Protestant as well as Catholic patients shall have the warm and helpful sympathy of a living faith.

Nurses and their Pay.

"Most of the nurses I know," says Charlotte Martin, "have come to the hospital from genuine love of the work, and a desire to help the struggling poor." That we are prepared to endorse as a general statement. "The reason," she continues, "why so few stay beyond three years is not that they are weary of well-doing, but that more lucrative prospects are opened up to them in private nursing. If the hospital authorities paid higher salaries, or allowed skilled nurses a percentage on the large fees paid by private patients, it is probable that there would be fewer changes in their nursing staff. As matters are at present there is no inducement for a woman who wishes to make nursing her profession to continue longer than three years in the hospital. Even if she eventually rose to be a matron her salary would be only about £60, with board and lodging; and the chances of such promotion are small." We have no sympathy with those nurses who have raised the recent outcry against hospitals, but we have great sympathy with this sober writer, who lets us know, not what the foolish and incompetent, but what the wise and good nurses are thinking. They are thinking that their work is hard, which is true; and they are thinking that their pay is small, which is also true. All fully certificated nurses are at least as old in years as the upper servants in private families. Are nurses equal or superior to the best sort of upper servants? They are certainly superior as a class, not only in birth and education, but in training and practical skill. Their work, too, is much more important. But is their pay any better? We doubt if, taking it all round, it is better: or if it be, it is very little. This is not right, and it is not wise. We do not wish to see all hospital nurses "ladies," but we do wish to see them all intelligent, disciplined, competent, and kindly women. In order to secure this, hospital nursing should be paid for at a rate in accordance with its intrinsic importance, and with the qualities demanded of those who follow it as a profession. There is a disposition, as Charlotte Martin is probably aware, on the part of a good many hospitals, both in London and the country, to indirectly raise the remuneration of their nurses by about one-fourth by paying a certain portion of the premium required to secure them an annuity in old age from the Royal National Pension Fund. If all the hospitals would do this an important advance would at once be made in the position of their nurses. The hospitals themselves would soon find that nurses would be less eager to leave them, when by leaving they diminished their prospect of a sufficient provision for their advancing years. We emphatically endorse the claim of the capable nurse to a much higher rate of pay; and we cannot but express the opinion that the very best method of improving the position of those women who nurse at hospitals is by making them all members of the Royal National, with the prospect of a pension sufficient for modest comfort when the time for hard work is past.

